# Supplementary material for: Genetic basis for coordination of meiosis and sexual structure maturation in Cryptococcus neoformans
Source: eLife. 2018 Oct 3;7:e38683. doi: 10.7554/eLife.38683 (PMC6235564; doi:10.7554/eLife.38683)
Supplement: Transparent reporting form [file elife-38683-transrepform.docx]

***eLife’s* transparent reporting form**

We encourage authors to provide detailed information *within their submission* to facilitate the interpretation and replication of experiments. Authors can upload supporting documentation to indicate the use of appropriate reporting guidelines for health-related research (see [EQUATOR Network](http://www.equator-network.org/%20)), life science research (see the [BioSharing Information Resource](https://biosharing.org/)), or the [ARRIVE guidelines](http://www.plosbiology.org/article/info:doi/10.1371/journal.pbio.1000412) for reporting work involving animal research. Where applicable, authors should refer to any relevant reporting standards documents in this form.

If you have any questions, please consult our Journal Policies and/or contact us: [editorial@elifesciences.org](mailto:editorial@elifesciences.org).

**Sample-size estimation**

- You should state whether an appropriate sample size was computed when the study was being designed
- You should state the statistical method of sample size computation and any required assumptions
- If no explicit power analysis was used, you should describe how you decided what sample (replicate) size (number) to use

Please outline where this information can be found within the submission (e.g., sections or figure legends), or explain why this information doesn’t apply to your submission:

This is not relevant to the study. No statistical methods were used to predetermine samples sizes and no size calculation was performed prior to experimental design.

**Replicates**

- You should report how often each experiment was performed
- You should include a definition of biological versus technical replication
- The data obtained should be provided and sufficient information should be provided to indicate the number of independent biological and/or technical replicates
- If you encountered any outliers, you should describe how these were handled
- Criteria for exclusion/inclusion of data should be clearly stated
- High-throughput sequence data should be uploaded before submission, with a private link for reviewers provided (these are available from both GEO and ArrayExpress)

Please outline where this information can be found within the submission (e.g., sections or figure legends), or explain why this information doesn’t apply to your submission:

Below are the details regarding how experiments were repeated:

Figure 1C-E, the results are from three biological replicates. This is stated in the legend of Figure 1.

Figure 2A, we performed 3 independent experiments. This is stated in the legend of Figure 2.

Figure 2B, > 50 cells in a given morphotype from three independent experiments were examined for mCherry-labelled proteins expression. This is stated in the legend of Figure 2.

Figure 2D-E, data shown are representative of three independent experiments conducted with similar results. This is stated in the legend of Figure 2.

Figure 3A-B, all RNA-seq data come from two independent experiments. This is stated in Materials and methods.

Figure 4B-D, all RNA-seq data come from two independent experiments. This is stated in Materials and methods.

Figure 4E, images are representative of more than five independent experiments conducted with similar results. This is stated in the legend of Figure 4.

Figure 5A, images are representative of more than five independent experiments conducted with similar results. This is stated in the legend of Figure 5.

Figure 5B-C, data are presented as the mean ± SD from six independent experiments. This is stated in the legend of Figure 5.

Figure 6A-B, data shown come from three independent experiments. This is stated in the legend of Figure 6.

Figure 6C, data are presented as the mean ± SD from three independent experiments. This is stated in the legend of Figure 6.

No data were excluded. The GEO accession number for the RNA-seq data reported in this study is GSE111975. This is stated in the Materials and methods section.

**Statistical reporting**

- Statistical analysis methods should be described and justified
- Raw data should be presented in figures whenever informative to do so (typically when N per group is less than 10)
- For each experiment, you should identify the statistical tests used, exact values of N, definitions of center, methods of multiple test correction, and dispersion and precision measures (e.g., mean, median, SD, SEM, confidence intervals; and, for the major substantive results, a measure of effect size (e.g., Pearson's r, Cohen's d)
- Report exact p-values wherever possible alongside the summary statistics and 95% confidence intervals. These should be reported for all key questions and not only when the p-value is less than 0.05.

Please outline where this information can be found within the submission (e.g., sections or figure legends), or explain why this information doesn’t apply to your submission:

We used two-tailed Student’s *t*-test to compare the mean florescence intensity or transcript levels from two groups. Fisher’s exact test was utilized to evaluate the significance of the overlap between two sets of genes. A two sided Kolmogorov-Smirnov test was used to verify the normality of the distribution of continuous variables. **P*-values < 0.05 were considered significant, and ****P*-values < 0.001 were considered very significant. In all figures, error bars represented mean ± standard deviation (SD) from at least three independent biological experiments. The detailed information can be found in the corresponding figure legends or in Materials and methods.

(For large datasets, or papers with a very large number of statistical tests, you may upload a single table file with tests, Ns, etc., with reference to sections in the manuscript.)

**Group allocation**

- Indicate how samples were allocated into experimental groups (in the case of clinical studies, please specify allocation to treatment method); if randomization was used, please also state if restricted randomization was applied
- Indicate if masking was used during group allocation, data collection and/or data analysis

Please outline where this information can be found within the submission (e.g., sections or figure legends), or explain why this information doesn’t apply to your submission:

This is not relevant to the study because specific genotype backgrounds were used for all the experiments and not applying a treatment to a larger subset of species.

**Additional data files (“source data”)**

- We encourage you to upload relevant additional data files, such as numerical data that are represented as a graph in a figure, or as a summary table
- Where provided, these should be in the most useful format, and they can be uploaded as “Source data” files linked to a main figure or table
- Include model definition files including the full list of parameters used
- Include code used for data analysis (e.g., R, MatLab)
- Avoid stating that data files are “available upon request”

Please indicate the figures or tables for which source data files have been provided:

Figure 3A-3B, supplementary file 1 (expression profiles of various developmental stages during unisexual reproduction and different gene sets identified based on gene induction timing through mating differentiation).

Source data files related to Figures 1C, 1D, 1E, 2A, 2D, 2E, 4A, 4C,5B, 5C, 5D, 6A, 6B, and Figure 1-supplement 1, Figure 4-supplement 2, Figure 6-supplements 1, 2A, 2B, 3 were provided.

.
